# Influence of the timing of brief, high-intensity cycling exercise on memory for novel words presented during an initial Portuguese lesson among older adolescents with ADHD: An exploratory study

**DOI:** 10.1371/journal.pone.0354741

**Published:** 2026-08-04

**Authors:** M. David Diggs, Daniel Ferreira da Silva, Robert Moser, Melissa J. McGranahan, Patrick J. O’Connor

**Affiliations:** 1 Department of Neurology, University of Alabama, Birmingham, Alabama, United States of America; 2 Department of Romance Languages, University of Georgia, Athens, Georgia, United States of America; 3 School of Medicine, Emory University, Atlanta, Georgia, United States of America; 4 Department of Kinesiology, University of Georgia, Athens, Georgia, United States of America; Soochow University, CHINA

## Abstract

This exploratory study examined whether brief high-intensity cycling exercise, performed either before or after a single online Portuguese language lesson, improved language acquisition or retention in older adolescents with ADHD, compared to a resting control condition. Participants (n = 29; mean age 19.2; no prior Portuguese knowledge) were randomly assigned to one of the three conditions: control, exercise before, and exercise after. The 29-minute Portuguese lesson introduced vocabulary, grammar, and syntax. Immediate learning (Day 1) and retention (Day 7) were assessed through sentence production, free recall, and recognition memory. The exercise groups completed three 30-second leg cycling sprints with active recovery. The language lesson and assessments were identical across conditions. Although no statistically significant differences were found, effect size analyses revealed that exercising before the lesson was associated with moderately better recognition memory on both Day 1 (d = 0.51) and Day 7 (d = 0.77), along with small improvements in free recall (d = 0.21 to 0.44). In contrast, exercising after the lesson was linked to worse recognition memory (d = –0.51) and fewer sentences created (d = –0.69 to –0.99) compared to control. The findings from this exploratory study show that in a sample of older adolescents with ADHD the brief, intense cycling exercise prior to an initial Portuguese lesson was associated with a pattern suggesting higher free-recall and recognition memory for Portuguese words, while the similar exercise performed after the language lesson was associated with a pattern suggesting worse memory performance on sentence production, a more complex cognitive task. Further research with larger and more diverse samples is needed to confirm the present findings and clarify optimal exercise timing during the initial and subsequent days of second language learning.

## 1. Introduction

On average a single bout of exercise transiently and modestly improves declarative memory [1–3]. A variety of factors have been hypothesized to moderate the magnitude of the improvements, including the timing of the exercise bout [4,5], the intensity of the exercise [6] and characteristics of the study participants such as their psychological health status and age [7]. For example, among children and adolescents with attention-deficit/hyperactivity disorder (ADHD), current evidence suggests that acute exercise does not alter short-term memory [8]. This conclusion is based on three experiments with ADHD children aged 8–14 whose memory was tested for non-language stimuli following a brief encoding period and tested from immediately to 10-minutes after moderate intensity exercise [9–11]. Multiple factors potentially account for the null effects of acute exercise on memory in these samples, including age, small sample bias, ADHD status, the moderate exercise intensity, and the timing of the exercise.

The timing of acute exercise, such as whether it occurs before or during memory encoding (when information is initially learned) or later when memories are becoming stabilized (memory consolidation phase), appears to moderate cognitive responses. An analysis of 25 acute exercise studies, for example, found that exercise performed prior to encoding improved memory by a small average magnitude (Cohen’s d effect size of 0.11, based on 66 effects) while exercise performed during early and late phases of memory consolidation (defined as less than or ≥ 4 hours after memory encoding, respectively) had moderate (d = 0.47, based on 66 effects) and large effects (d = 1.05, based on 3 effects) [4]. This analysis showed a clear advantage of timing exercise after encoding. A separate meta-analysis found that exercise after encoding compared to exercise prior to encoding induced larger mean effects for recognition memory (Cohen’s d = 0.60 vs. −0.06) but not for free-recall memory (d = 0.19 vs. d = 0.40), concluding that exercise timing has differential effects on different aspects of episodic memory [5].

The intensity of a single bout of exercise may be a moderator of the cognitive responses. Systematic reviews have drawn different conclusions based in part on including different investigations and analyzing the data in different ways. For example, an early review found that post-exercise memory was best following low-intensity exercise [2], subsequent reviews found better memory performance after vigorous exercise [4,6], and the most recent review concluded no moderator effect of exercise intensity [3]. It is plausible that the relationship between memory and the intensity of acute exercise is not straight forward. For instance, there is emerging evidence that exercise intensity interacts with other relevant variables, such as the post-exercise recovery time, to moderate cognitive responses [12].

While the idea that cognitive processes are influenced by bodily states dates back centuries, the 21^st^ century has brought an acceleration of research and theory linking classic ideas about embodied cognition to practical problems such as second language learning (2LL) [13]. Relevant here are several experiments which found that the addition of regular physical activity to weeks-long language learning programs enhanced aspects of 2LL [14–16]. A clear gap in knowledge is the time course of such beneficial effects. For example, do these effects occur during the first day of 2LL or do such benefits take weeks to accrue? Thus, whether or not the first exercise bout impacts initial 2LL would be worth knowing for both practical and academic reasons (e.g., potential implication regarding the biological mechanisms for the effect). While many of the prior studies of acute exercise on cognition have included memory for words in the native language of the study participants as part of the outcome assessments, it appears that only a few have focused on memory for novel words such as when individuals are engaged in 2LL. The pioneering study appears to have been conducted by Winter and colleagues [17]. In a sample of young adult German males (n = 27) the influence of moderate intensity (40-minutes) and high intensity (2 x 3-min bouts) running was examined for the immediate and long-term effects on the associative learning of picture-pseudo word pairs presented briefly (1 second). There was a 20% improvement in associative learning after the high intensity running bouts compared to control. These effects were maintained at the 1- and 8-week follow ups. In a later study of acute exercise and 2LL Liu et al. tested 40 Chinese adolescents with basic English proficiency [18]. Compared to a non-exercise control, a bout of moderate intensity cycling exercise performed during 5-second exposures to novel word-picture pairs resulted in improved recognition memory for the words as well as enhanced recognition of correct sentences that had not been an explicit part of the novel word-picture pair language exposure. These effects were maintained at a 4-week follow up. The authors concluded that physical activity improves 2LL “not only at the specific level of training (i.e., lexical level), but also at a more general, untrained level of processing (i.e., sentence level)”.

While this small corpus of research supports that a single bout of exercise can enhance 2LL, there remains uncertain generalizability of the finding to other samples such as older adults and neurodivergent individuals. If a single bout of exercise does promote 2LL then it would be useful to know if the effect extends to those with learning challenges such as individuals with ADHD. A large body of literature supports that people with ADHD have greater difficulties with learning but the evidence regarding 2LL is limited according to a systematic review published in 2025 [19].

Here we tested, compared to a resting control condition, whether brief high-intensity cycling exercise timed before or after engagement in a single initial online Portuguese language class improved the immediate acquisition, or the long-term retention, of the Portuguese lesson in a sample of older adolescents with ADHD. Novel features of the experiment included the older adolescent ADHD sample, a longer time for memory encoding (a 29 minute 2LL lesson), and testing memory for free-recall as well as sentence creation and recognition memory. Based on the extant prior research briefly summarized above and largely conducted with neurotypical adults [4] it was hypothesized that, compared to a resting control condition, recognition memory performance would be best in the “Exercise After” condition. This exploratory study assessed the direction and magnitude of effect sizes in order to suggest whether larger-scale research is warranted.

## 2. Materials and methods

### 2.1. Research design

A randomized posttest only experimental research design with two intervention groups and one control group was used. This research design was chosen to maintain the internal validity of the experiment by eliminating the potential reactive or interactive effects of conducting Portuguese language pretesting [20]. Pretest language scores could reasonably be assumed to be homogenous among the groups because a lack of Portuguese language proficiency was a prerequisite to participation.

Participants were randomly assigned to one of three conditions: a no exercise resting control (CON) condition, an exercise before a second language learning lesson (ExB4) condition, or an exercise after the language learning lesson (ExAFTR) condition. Research Randomizer (www.randomizer.org) was used by PJO to generate the random allocation sequence. PJO then created envelopes with the allocations and labelled them (i.e., participant 001, participant 002, participant 003, etc.…). These were stored in a locked office and later opened by MDD and used when a participant was ready to be allocated on testing day 1.

### 2.2. Recruitment

The study was conducted in a manner consistent with the Declaration of Helsinki and approved by the local Institutional Review Board (PROJECT00001464, approved January 2020). Participants were recruited via emails, listserv announcements, flyers on bulletin boards, in-class presentations and snowball sampling within the context of a large public University setting. The University is located in a medium-sized urban area in the Southeastern United States. Individuals potentially interested in participating initially were directed to complete online screening questions to determine eligibility. Excluded were those: (i) younger than the age of 15 and those older than the age of 24, (ii) who reported the ability to read, speak or understand any level of Portuguese, (iii) without Attention Deficit Hyperactivity Disorder (ADHD), and (iv) for whom brief, high-intensity leg cycling exercise was contraindicated based on a yes response to any of the seven general health questions of the Physical Activity Readiness for Everyone tool; an exception was made for those listing ADHD as a chronic medical condition [21].

Actually recruited were female and male older adolescents, aged 18–21 years, primarily with a self-reported current diagnosis of ADHD (n = 27). Two individuals without a current diagnosis but with probable ADHD were included based on elevated scores on the Adult ADHD Self-Report Screening Scale for DMS-5 (ASRS-5) [22]. The ASRS-5 is a widely used tool that closely reproduces evaluations made by well-trained clinicians. The sensitivity and specificity of the ASRS-5 cut score used has been reported to be 91.4 and 96.0, respectively [23]. Sensitivity analyses revealed that the statistical significance of the findings as well as the conclusions drawn here were identical whether these two individuals were included or excluded, so data from the two were included.

Data from a total of 15 females and 14 males were included in the analysis, participation starting 7/1/2021 and ended on 7/27/2023. Participants were provided online gift cards of $20 and $10, respectively for completing the testing on Day 1 and Day 7. An overview of key elements of the investigation are presented in Table 1.

**Table 1:** Selected key elements and overall structure of the investigation.

	Day 1 Testing				Day 7 Testing
Conditions	17-minutes before the language condition	29-minute language learning condition	17-minutes after the language condition	Short-term language acquisition testing	Long-term language retention testing
Control	Seated quiet rest, no cell phone access	Language learning lesson	Seated quiet rest, no cell phone access	Free recall and recognition memory assessment	Free recall and recognition memory assessment
Exercise before language lesson	High intensity exercise bouts	Language learning lesson	Seated quiet rest, no cell phone access	Free recall and recognition memory assessment	Free recall and recognition memory assessment
Exercise after language lesson	Seated quiet rest, no cell phone access	Language learning lesson	High intensity exercise bouts	Free recall and recognition memory assessment	Free recall and recognition memory assessment

### 2.3. Day 1 testing

Most of the testing occurred during the COVID-19 pandemic which started in March of 2020 and ended in May of 2023. During a brief initial meeting, held virtually using cell phones, participants were provided with a description of the study and shown the laboratory, including the safety precautions to be taken (e.g., air filtration system). At the end of the virtual meeting, the date and time of day 1 testing were scheduled. To be tested the participants also were required to report freedom from COVID-19 symptoms, including a fever based on body temperature measurements taken with a forehead temporal artery thermometer prior to entering the lab on both testing days.

Participants were asked to accurately describe the study requirements to a researcher based on the prior virtual meeting after which clarifications were made as needed and questions about the study were addressed. This process involved individuals reading and signing the informed consent document, which had been reviewed and approved by one of the University’s Institutional Review Boards (PROJECT00001464).

A chest strap was used to assess heart rate (Polar; Polar Electro Inc., Lake Success, NY). Height and body mass were measured to the nearest cm and 0.5 kg using a calibrated balance beam scale (Detecto Model 439). Participants sat at a desk and completed the 20-item state anxiety portion (Y-1) of the State-Trait Anxiety Inventory [24]. Resting systolic and diastolic blood pressures were measured oscillometrically using an automated Omron BP760N Blood Pressure Monitor. The Omron BP760N is accurate to ± 3 mmHg [25].

### 2.4. Portuguese lesson

Participants sat alone in a laboratory room in front of a computer, without access to their cell phone which had been moved to a different room, and watched, engaged with and completed requested assignments that were incorporated into a single online video (a Portuguese language lesson for beginners). The 29-minute lesson was created by Portuguese language experts with years of experience engaged in Portuguese language pedagogy (DFdS and RM). Using a checklist, a researcher confirmed every 4 minutes that participants were engaged in the assignments or attending to the video; for example, in no instance was a participant obviously not attending (e.g., turned away from the screen, eyes closed or napping).

In brief, the start of the lesson showed an illustration of an elementary school classroom with 16 common elements in the image, initially all identified using English words (e.g., a book, a student, a backpack). Participants were given 2 minutes to look at the image. Next, the identical classroom image was shown again with the same 16 elements but this time the elements were identified using Portuguese words. An associated audio played, with the unseen instructor (DFdS) correctly pronouncing each word and introducing the concept of feminine and masculine forms of an article preceding each word (i.e., um/uma[both translate as a or an]). Participants were given 2 minutes to review the slide and were told to write words on a paper provided (take notes) if it would be helpful to aid in remembering the words. Most participants took some notes (n = 22/29). Next, images of 7 different classroom elements were presented separately on the bottom of a slide and 7 associated Portuguese words were presented at the top of the slide. Participants were given 2 minutes and instructed to accurately match the 7 Portuguese words to the 7 images and write the answers on paper. Then a review of the correct matches was provided by the video lesson instructor. Next, 4 color words (i.e., vermelho [red], amarelo [yellow], marrom [brown] and azul [blue]) were introduced and 2 minutes were again given to review the 16 classroom elements and note which elements were characterized by the 4 colors. Next, the participants were asked to correctly identify both the Portuguese words for several elements in the image and the color words describing those elements. Next, a conjugated verb (i.e., há [there is/there are]) as well as feminine and masculine forms of an article (i.e., um/uma) were presented and reviewed. Next, the participants were asked to fill in blanks using two word phrases shown at the top of a slide to make short sentences that described a few of the image elements depicted (e.g., há um ___ ____; one example of a correct answer was: há um livro vermelho). The video lesson can be viewed here: https://drive.google.com/file/d/1lAKUXJop7rXEOvBgyKdxKXDz1cMgilE3/view.

### 2.5. Exercise and control conditions

The ExB4 and ExAFTR conditions were identical except for their timing. In the ExB4 condition the 17-minute exercise session was completed immediately prior to the language lesson (before memory encoding). In the ExAFTR condition the 17-minute exercise session was completed immediately after (within 1-minute) the language lesson (during the early memory consolidation phase).

The exercise sessions included a warm-up followed by 3 brief high-intensity leg cycling exercise bouts. The exercise sessions were performed outdoors for safety reasons because of the COVID-19 pandemic for most study participants; these were conducted in a semi-secluded tree-lined area next to an exterior laboratory door. The exercise sessions were rescheduled as needed to avoid extreme weather conditions. The exercise was completed using a Lode (Excalibur) ergometer. The high intensity exercise intervals consisted of 3 x 30-sec all-out leg sprints against a high resistance (0.085 kilograms resistance to the flywheel per kilogram body weight). The resistance was calculated and applied using computer software (Wingate Lode program, version 1.0.11). Participants warmed up for 5-minutes at a low intensity (25–50 W). Approximately 10-sec before the resistance increased, participants were instructed to begin pedaling faster. Once full resistance was applied, participants were verbally encouraged in a standard fashion by the test administrator to give a maximal effort throughout the entire 30-sec sprint. Participants then pedaled at a low resistance of 25–50 W for 4-minutes of active recovery. This protocol of sprint and recovery was repeated two additional times. Immediately after each sprint, ratings of perceived exertion were obtained using the 6–20 Borg scale [26]. Standardized instructions regarding the scale were provided prior to the warm-up. The participants completed 2.5 minutes of cool down; thus, the entire exercise session lasted 17-minutes.

The CON condition involved sitting alone in a chair in the laboratory without the use of a cell phone or computer for 17-minutes prior to or after the language lesson.

### 2.6. Day 7 testing

Participants entered the laboratory, sat at a desk and were given 2-minutes to write down as many of the Portuguese words presented in the prior week’s lesson as they could remember. Next, 2-minutes were given to write down as many simple Portuguese sentences as they could construct. Participants were then given 3-minutes to review a list of 20 words presented on a piece of paper and identify any that were presented in the prior week’s lesson. The list included 10 words that were included in the language lesson and 10 that were not. The words used in the word recognition tasks on Day 1 and Day 7 are provided in Tables 2 and 3, respectively.

**Table 2:** List of word used in the word recognition test on Day 1. Participants were instructed to circle only the words that had been presented in the lesson. The bolded words below were those presented in the lesson.

vermelho	relógio	cardeira	carteado
aula	compulsação	quatro	alemão
diz	chinela	marroada	**mesa**
**televisão**	**amarelo**	**azul**	papéis
pagador	**há**	**livro**	**caderno**

**Table 3:** List of words that were used in the word recognition test on Day 7. Participants were instructed to circle the words that had been presented in the lesson. The bolded words below were those presented in the lesson.

vereamo	lógico	cardada	carteira
**aluna**	**computador**	**quadro**	**aluno**
**giz**	**mochila**	**marrom**	massa
teleférico	amabil	azado	**lápis**
**apagador**	hábia	livre	cadeo

### 2.7. Primary outcomes

The primary outcomes were measured during testing on both Day 1 and Day 7. The testing of primary outcomes was identical for each condition and on both Days with one exception – when the primary outcomes were measured following exercise, the participants were given time to towel off sweat, take a drink of water and walk less than 15 seconds to the testing room. The primary outcome testing was initiated approximately 1-minute after the language lesson in the control and ExB4 conditions and about 1-minute after exercise in the ExAFTR condition.

*Free-recall memory.* The participants were given 2-minutes to “write down as many of the Portuguese words presented in the lesson that you can remember”. A total of 23 Portuguese words were covered in the language lesson. Participants next were instructed to: “Write three simple sentences using the information you learned in the Portuguese lesson”. Each sentence was graded (by DFdS) and scored as the number of correct sentences with no significant mistake (range = 0–3). The free recall of words were graded in three ways: the number of accurate words (with no errors; range = 0–23), the number of discernable words (judged as Portuguese words but some with minor mistakes; range = 0–23), and an overall grade (scored blindly by DFdS), rated 0–2 as follows: 0 = no words written or all the words written were inaccurate, 0.5 = less than half of the words were accurate, 1 = half the words were correct but some were not discernable, 1.5 = 2–3 mistakes and all words discernable, 2 = no mistakes or only one minor mistake.

*Recognition memory.* After the free recall memory test, the participants were given a sheet of paper that listed 20 words. About half of the words (n = 9–11) had been presented in the Portuguese lesson. The other words were “lure” words (n = 10–11). That is, the words were either Portuguese words not presented in the lesson or words created to appear to be Portuguese words even though they were actually pseudo-words. Participants were given 3-minutes to indicate which words on the list had been presented in the lesson. A researcher (DFdS) blindly scored this test for the number of words on the list that were correctly identified as having been presented in the lesson (aka., hits).

### 2.8. Other measurements

*Heart rate.* Heart rate was assessed to document exercise intensity. A Polar chest strap measured the electrocardiogram and heart rate per minute was continuously displayed on a Polar watch. Heart rate data were recorded before and immediately after each exercise bout and the equivalent time periods during the CON condition.

*Perceived exertion.* Ratings of perceived exertion (RPE) were used to measure the perceived physical effort put into the exercise and CON conditions. RPE was assessed, using the 6–20 RPE scale, before and immediately after each exercise sprint interval and the equivalent time periods during the CON condition.

*State anxiety.* Elevated levels of anxiety have been shown to negatively influence second language learning [27] and acute exercise often reduces anxiety [28]. Accordingly, state anxiety, assessed using the 20-item state portion (Y-1) of the State-Trait Anxiety Inventory, was measured 17-minutes before and 17-minutes after the language lesson.

Number of proficient languages. Participants were asked how many languages they were proficient in beyond English.

### 2.9. Statistical analysis

Acquired data were entered into a spreadsheet and double checked for accuracy. All statistics were performed using IBM SPSS Statistics Version 29.0.1.0. Data were examined visually and statistically for outliers, normality and homogeneity of variance. One variable, percentage hit accuracy on Day 1 testing, was not normally distributed. Analysis of the raw scores and square root transformed data yielded identical statistical results for this variable.

Differences among the groups in response to the short-term acquisition effects (Day 1) were tested using one-way ANOVAs. Long-term retention effects were tested using ANCOVA on Day 7 scores adjusting for Day 1 scores as the covariate. ANCOVA model assumption (e.g., homogeneity of regression slopes) were tested (e.g., examining the Group × Post 1 interaction) and met. The number of proficient languages and baseline state anxiety were included as covariates to test if these variables moderated the effect of exercise on memory; none of these ANCOVA models changed the statistical significance of the results. Mixed model ANOVAs tested the Group, Time (3 exercise bouts across time) and interaction effects for the exercise data (heart rate, RPE). The Greenhouse–Geisser adjustment to degrees of freedom was used when the sphericity assumption was not met in the mixed model ANOVAs. Sample sizes for females and males were inadequate for a statistically powerful test of sex effects, so sex was not included as a covariate. Non-trivial effect size differences between the exercise and CON groups (> 0.20) are provided as Cohen’s d and conventional guidelines index d = 0.20, d = 0.50 and d > 0.80 as small, moderate and large sized effects. Because analyses were exploratory and intended to generate hypotheses, adjustments for multiple comparisons were not applied as has been suggested previously [29,30]. Exact p-values, effect sizes, and confidence intervals are reported and findings should be interpreted cautiously.

## 3. Results and analysis

### 3.1. Sample characteristics

The study participants (n = 29) were normotensive (mean blood pressures of 115/75 mmHg) and older adolescents (mean age of 19 years) with a healthy body mass (mean BMI of 22.6 kg/m^2^). The median number of second language proficiencies was 1 other language (range of 0–9). The mean state anxiety values were within the age-level norms. The descriptive data for these variables are summarized in Table 4 by study group. There were no statistically significant differences between the study groups on these variables.

**Table 4:** Characteristics of the three study groups (mean ± standard deviation).

	No Exercise Control (n = 10)	Exercise Before (n = 7)	Exercise After (n = 12)
Age (years)	19.3 ± 1.3	19.3 ± 1.3	19.2 ± 1.1
Female: Male (n:n)	5:5	4:3	6:6
Height (centimeters)	175.5 ± 9.8	171.4 ± 6.4	173.9 ± 10.7
Weight (kilograms)	69.6 ± 9.2	64.3 ± 9.1	69.4 ± 10.7
Body mass index (kg/meters²)	22.6 ± 9.5	21.9 ± 7.8	22.9 ± 10.7
Systolic pressure (mmHg)	112.8 ± 11.8	116.6 ± 17.4	116.1 ± 14.7
Diastolic pressure (mmHg)	72.1 ± 10.3	79.0 ± 7.6	74.3 ± 7.8
Other language proficiency (n)	2.0 ± 2.5	0.7 ± 0.8	0.9 ± 0.9
State anxiety – pre (20–80)	34.7 ± 10.0	40.7 ± 6.5	37.3 ± 10.6
State anxiety – post (20–80)	33.8 ± 10.8	39.6 ± 8.0	37.5 ± 10.2

### 3.2. Heart rate, perceptual effort and anxiety responses to the conditions

State anxiety did not change significantly in response to the control or exercise conditions.

In the CON condition (n = 10), the lowest possible RPE were reported and the mean resting heart rate remained stable during the testing session with an overall mean of 82 ± beats/min. In the exercise conditions, heart rates increased in response to exercise and the mean heart rates ranged from 154 to 178 beats/min immediately after the cycling sprints. For the immediate post-sprint heart rate data, ANOVA revealed insignificant Group (F(_1,17_) = 1.503, η²p = 0.007, p < 0.735) and Group x Time (F(_1.627, 27.653_) = 0.631, η²p = 0.036, p < 0.508) effects while the Time main effect was significant (F(_1.627, 27.653_) = 25.273, η²p = 0.598, p < 0.001). Mean heart rates increased with each successive sprint for both exercise groups. Mean RPE immediately after the cycling sprints ranged from 13.0 to 16.6 raw score units. ANOVA revealed an insignificant Group x Time effect (F_(1.566, 26.672)_ = 0.187, η²p = 0.011, p = 0.77) with significant Time (F_(1.566, 26.672)_ = 5.721, η²p = 0.252, p = 0.013) and Group (1,17, F_(1,17)_ = 4.903, η²p = 0.224, p < 0.001) main effects. Mean RPE increased with each successive sprint for both exercise groups, and the overall mean ratings were higher for the ExB4 group (mean RPE = 15.9, closest verbal anchor “hard”) compared to the ExAFTR group (mean RPE = 13.5, closest verbal anchor “somewhat hard”). The heart rate and perceived exertion data in the CON, ExB4 and ExAFTR conditions are provided in Table 5.

**Table 5:** Heart rate and perceived exertion before and immediately after each sprint in the exercise conditions and during the seated resting control condition (mean ± standard deviation).

	No Exercise Control (n = 10)	Exercise Before (n = 7)	Exercise After (n = 12)
Heart Rate (beats/min)			
**Sprint 1**			
Before	83.0 ± 5.2	107.0 ± 27.4	108.1 ± 22.5
Immediately after	81.9 ± 8.2	154.0 ± 31.7	165.5 ± 8.3
**Sprint 2**			
Before	82.0 ± 8.3	128.9 ± 25.8	123.5 ± 15.5
Immediately after	83.4 ± 7.1	161.7 ± 33.2	175.9 ± 7.9
**Sprint 3**			
Before	81.7 ± 7.9	135.7 ± 26.8	132.6 ± 16.7
Immediately after	83.0 ± 7.3	168.1 ± 34.3	178.1 ± 10.0
Perceived Exertion (6–20)			
**Sprint 1**			
Before	6.1 ± 0.3	7.6 ± 1.1	7.9 ± 1.5
Immediately after	6.0 ± 0.0	15.1 ± 1.3	13.0 ± 2.9
**Sprint 2**			
Before	6.0 ± 0.0	8.6 ± 1.8	8.9 ± 1.6
Immediately after	6.0 ± 0.0	15.9 ± 1.9	13.4 ± 2.3
**Sprint 3**			
Before	6.0 ± 0.0	10.1 ± 1.7	8.9 ± 1.9
Immediately after	6.0 ± 0.0	16.6 ± 2.1	14.0 ± 2.8

### 3.3. Short-term (Day 1) language acquisition

Free-recall of words

Table 6 provides a summary of the four free-recall results: discernable words, accurate words, overall graded score and comprehensible sentences.

**Table 6:** Free recall memory test results by experimental day and group (mean ± standard deviation, and 95% confidence interval [95% CI]).

	Controls (n = 10)	Exercise Before (n = 7)	Exercise After (n = 12)	ExB4 – CON ES 95% CI	ExAFTR – CON ES 95% CI
**Word recall**					
Discernible words (0–23 range)					
Day 1	15.1 ± 4.9	17.0 ± 3.1	13.8 ± 4.7	0.44 −0.53, 1.42	−0.27 −1.11, 0.57
Day 7	9.3 ± 5.5	10.6 ± 5.0	9.5 ± 5.7	0.25 −0.72, 1.21	0.03 −0.80, 0.87
D7 minus D1	−5.8 ± 2.3	−6.4 ± 3.7	−4.3 ± 4.3	0.20 −1.17, 0.76	0.42 −0.43, 1.27
Accurate words (0–23)					
Day 1	11.8 ± 6.3	13.3 ± 3.6	11.3 ± 5.1	0.28 −0.69, 1.25	−0.09 −0.93, 0.75
Day 7	6.9 ± 6.1	8.1 ± 5.2	7.1 ± 5.4	0.21 −0.76, 1.18	0.04 −0.80, 0.87
D7 minus D1	−4.9 ± 3.0	−5.1 ± 3.8	−4.2 ± 4.3	−0.06 −1.03, 0.91	−0.19 −0.66, 1.03
Word grade (0–2)					
Day 1	1.4 ± 0.5	1.3 ± 0.3	1.4 ± 0.6	−0.23 −1.20, 0.74	0.00 −0.84, 0.84
Day 7	1.0 ± 0.7	1.2 ± 0.6	1.0 ± 0.6	0.30 −0.70, 1.27	0.00 −0.84, 0.84
D7 minus D1	−0.4 ± 0.7	−0.1 ± 0.5	−0.4 ± 0.5	0.48 −0.50, 1.46	0.00 −0.84, 0.84
**Comprehensible sentences (0–3)**					
Day 1	3.0 ± 0.0	2.6 ± 1.1	2.2 ± 1.1	−0.57 −1.56, 0.41	−0.99 −1.87, −0.09
Day 7	2.1 ± 1.0	1.3 ± 1.3	1.9 ± 1.0	−0.71 −1.70, 0.29	−0.20 −1.04, 0.64
D7 minus D1	−1.0 ± 1.0	−1.3 ± 1.3	−0.8 ± 1.2	0.27 −1.24, 0.70	−0.18 −0.66, 1.02

ExB4 = Exercise before condition; ExAFTR = Exercise after condition; CON = Control condition; ES = Cohen’s d effect size; 95% CI = 95% confidence interval.

The total number of discernable words recalled on Day 1 was insignificantly different among the CON (mean = 15.1 words), ExB4 (17.0 words) and ExAFTR (13.8 words) groups (F_(2,26)_ = 1.182, η²p = 0.083, p = 0.323). The ExB4 group was moderately better (d = 0.44) and the ExAFTR was slightly worse (d = −0.27) compared to CON.

The mean ± SD total number of words accurately recalled on Day 1 was insignificantly different among the CON (11.8 ± 6.3 words), ExB4 (13.3 ± 3.6 words) and ExAFTR (11.3 ± 5.1 words) groups (F_(2,26)_ = 2.174, η²p = 0.143, p = 0.134). The ExB4 group was slightly better (d = 0.28) compared to CON.

The mean ± SD grade score for free recall of words on Day 1 was insignificantly different among the CON (1.35 ± 0.47), ExB4 (1.29 ± 0.27) and ExAFTR (1.42 ± 0.60) groups (F_(2,26)_ = 0.338, η²p = 0.025, p = 0.716). The ExB4 group was slightly worse (d = −0.16) and the ExAFTR was slightly better (d = 0.12) compared to CON.

The ANOVA for the total number of comprehensible sentences written was insignificant (F_(2,26)_ = 2.174, η²p = 0.143, p = 0.134). An independent t-test (t_(20)_ = 2.318, p = 0.031) showed that the ExAFTR wrote significantly fewer sentences compared to the CON (2.2 versus 3.0). The magnitude of this effect was large (d = −0.99). There was no difference between the CON and the ExB4 (3.0 vs 2.6 sentences) group (t_(15)_ = 1.213, p = 0.244).

Recognition memory

Table 7 provides a summary of the recognition memory results. Participants overall (n = 29) showed good recognition memory performance for hits (90.8 ± 14.9% accuracy). The percentage of words accurately recognized (hits) was insignificantly different among the CON (93.3%), ExB4 (96.8%) and ExAFTR (85.2%) groups (F_(2,26)_ = 1.650, η²p = 0.113, p = 0.212). The ExB4 group had moderately better recognition (d = 0.51) and the ExAFTR had moderately worse recognition (d = −0.51) compared to CON.

**Table 7:** Recognition memory (mean ± SD) for Portuguese words during acquisition (Day 1 data) and retention (Day 7 data) phases as a function of study group (mean ± standard deviation) and Cohen’s d effect sizes and 95% confidence intervals.

	Controls (n = 10)	Exercise Before (n = 7)	Exercise After (n = 12)	ExB4 – CON ES 95% CI	ExAFTR – CONES 95% CI
**Day 1**					
Hits (%)	93.3 ± 5.7	96.8 ± 8.4	85.2 ± 20.9	0.51 −0.47, 1.49	−0.51 −1.36, 0.35
**Day 7**					
Hits (%)	86.0 ± 15.8	95.7 ± 5.3	86.7 ± 16.7	0.76 −0.24, 1.76	0.04 −0.80, 0.88
D7 minus D1	−7.3 ± 17.1	−1.1 ± 11.4	1.5 ± 17.2	0.41 −0.56, 1.39	0.51 −0.34, 1.37

ExB4 = Exercise before condition; ExAFTR = Exercise after condition; CON = Control condition; ES = Cohen’s d effect size; 95% CI = 95% confidence interval.

For descriptive purposes, Table 8 provides the percentage correct on recognition memory for each of the 23 words presented in the language lesson (target words) and the 21 lure words included in the recognition memory test but not presented in the lesson. Seven words lured at least 1 control into the false claim of recognizing it as a word that was presented in the lesson. Four of these effective lure words were quite similar to words presented in the lesson (e.g., quatro versus quadro). Two target words (i.e., presented in the lesson) that were included in the recall test on Day 7 were similar enough to each other to create a memory challenge (aluno and aluna), especially for the CON.

**Table 8:** The percentage correct on recognition memory for each of the 23 words presented in the language lesson (targets) and the 21 lure words included in the recognition memory test but not presented in the lesson by experimental day and group, and non-trivial Cohen’s d effect sizes and 95% confidence intervals.

		Controls (n = 10)	Exercise Before (n = 7)	Exercise After (n = 12)	ExB4 vs CON ES 95% CI	ExAFTR vs CON ES 95% CI
**Targets (Day 1)**	**English meaning**					
1. vermelho	red	100 ± 0.0	100 ± 0.0	100 ± 0.0		
2. amarelo	yellow	100 ± 0.0	100 ± 0.0	91.7 ± 28.9		
3. há	there is	100 ± 0.0	100 ± 0.0	83.3 ± 38.9		
4. azul	blue	100 ± 0.0	100 ± 0.0	91.7 ± 28.9		
5. livro	book	100 ± 0.0	100 ± 0.0	91.7 ± 28.9		
6. mesa	table	100 ± 0.0	100 ± 0.0	83.3 ± 38.9		
7. televisão	television	100 ± 0.0	85.7 ± 37.8	75.0 ± 45.2	−0.60 −1.58, 0.39	−0.75 −1.61, 0.12
8. relógio	clock	90.0 ± 31.6	100 ± 0.0	100 ± 0.0	0.41 −0.57, 1.38	0.47 −0.38, 1.32
9. caderno	notebook	50.0 ± 52.7	85.7 ± 37.8	50.0 ± 52.7	0.75 −0.24, 1.75	
**Lure words not presented in lesson (Day 1)**	**Similar Portuguese word in lesson**					
aula	aluna	0 ± 0.0	0 ± 0.0	0 ± 0.0		
diz	giz	0 ± 0.0	0 ± 0.0	0 ± 0.0		
compulsação	computador	0 ± 0.0	0 ± 0.0	0 ± 0.0		
chinela	mochila	0 ± 0.0	0 ± 0.0	0 ± 0.0		
papéis	lápis	0 ± 0.0	0 ± 0.0	0 ± 0.0		
alemão	amarelo	0 ± 0.0	0 ± 0.0	0 ± 0.0		
morada	mochila	0 ± 0.0	0 ± 0.0	8.3 ± 28.9		
carteado	caderno	10.0 ± 31.6	0 ± 0.0	0 ± 0.0	−0.41 −1.38, 0.57	−0.47 −1.32, 0.38
pagador	apagador	10.0 ± 31.6	0 ± 0.0	8.3 ± 28.9	−0.41 −1.38, 0.57	−0.06 −0.90, 0.78
quatro	quadro	20.0 ± 42.2	42.9 ± 53.5	8.3 ± 28.9	0.49 −0.49, 1.47	−0.33 −1.17, 0.52
cardeira	carteira	80.0 ± 42.2	57.1 ± 53.4	50.0 ± 52.2	−0.49 −1.47, 0.49	−0.63 −1.48, 0.23
**Targets (Day 7)**	**English meaning**					
10. computador	computer	100 ± 0.0	100 ± 0.0	100 ± 0.0		
11. lápis	pencil	100 ± 0.0	100 ± 0.0	100 ± 0.0		
12. mochila	backpack	100 ± 0.0	100 ± 0.0	91.7 ± 28.9		
13. giz	chalk	100 ± 0.0	100 ± 0.0	91.7 ± 28.9		
14. marrom	brown	100 ± 0.0	85.7 ± 37.8	91.7 ± 28.9		
15. carteira	desk	90.0 ± 31.6	85.7 ± 37.8	75.0 ± 45.2	−0.13 −1.09, 0.84	−0.38 −1.22, 0.47
16. apagador	eraser	80.0 ± 42.2	100 ± 0.0	91.7 ± 28.9	0.61 −0.38, 1.60	0.33 −0.52, 1.17
17. quadro	board	70.0 ± 48.3	85.0 ± 37.8	66.7 ± 49.2	0.34 −0.63, 1.31	
18. aluno	male student	60.0 ± 51.6	100 ± 0.0	91.7 ± 28.9	1.00 −0.02, 2.02	0.78 −0.09, 1.65
19. aluna	female student	60.0 ± 51.6	100 ± 0.0	66.7 ± 49.2	1.00 −0.02, 2.02	0.13 −0.71, 0.97
**Lure words** **(Day 7)**	**Similar Portuguese word in lesson**					
teleférico	televisão	0 ± 0.0	0 ± 0.0	0 ± 0.0		
amabil	amarelo	0 ± 0.0	0 ± 0.0	0 ± 0.0		
acordada	caderno	0 ± 0.0	0 ± 0.0	0 ± 0.0		
assada	aluna	0 ± 0.0	0 ± 0.0	0 ± 0.0		
verador	pagador	0 ± 0.0	0 ± 0.0	16.7 ± 39.0		
massa	mesa	0 ± 0.0	14.3 ± 37.8	8.3 ± 28.9		
cadeado	caderno	0 ± 0.0	14.3 ± 37.8	8.3 ± 28.9		
lógico	relógio	10.0 ± 31.6	0 ± 0.0	0 ± 0.0	−0.41 −1.38, 0.57	−0.47 −1.32, 0.38
hábia	há	10.0 ± 31.6	0 ± 0.0	8.3 ± 28.9	−0.41 −1.38, 0.57	−0.06 −0.90, 0.78
livre	livro	60.0 ± 51.6	85.7 ± 37.8	83.3 ± 38.9	0.55 −0.43, 1.54	0.52 −0.34, 1.37
**In lesson, not tested words**	**English meaning**					
20. professor	professor					
21. video	video					
22. um	a/an masculine					
23. uma	a/an feminine					

Seven words lured at least 1 control into the false claim of recognizing it as a word that was presented in the lesson (highlighted in yellow); 4 of these effective as lure words were quite similar to words presented in the lesson (highlighted in blue). Two target words (i.e., presented in the lesson) that were included in the recall test on Day 7 were similar enough to each other to create a memory challenge, especially for the controls (highlighted in green).

### 3.4. Long-term (7-day) language retention

Free-recall of words

Table 6 provides a summary of the four free-recall results: discernable words, accurate words, overall graded score and comprehensible sentences.

The total number of discernable words recalled on Day 7 was insignificantly different among the CON (mean = 9.3 words), ExB4 (10.6 words) and ExAFTR (9.5 words) groups (F_(2,26)_ = 0.122, η²p = 0.009, p = 0.885). The ExB4 group was slightly better (d = 0.25) compared to CON. Across all three groups (n = 29), fewer total discernable words were recalled on Day 7 (9.7 ± 5.3) compared to Day 1 (15.0 ± 4.5). The Day 7 scores were insignificantly different across groups when using Day 1 scores as a covariate (F_(2,25)_ = 0.729, η²p = 0.055, p = 0.492).

The total number of accurate words recalled on Day 7 was insignificantly different among the CON (mean = 6.9 words), ExB4 (8.1 words) and ExAFTR (7.1 words) groups (F_(2,26)_ = 0.112, η²p = 0.009, p = 0.894). The ExB4 group was slightly better (d = 0.12) compared to CON. Across all three groups (n = 29), fewer total accurate words were recalled on Day 7 (7.3 ± 5.5) compared to Day 1 (11.9 ± 5.1). The Day 7 scores were insignificantly different across groups when using Day 1 scores as a covariate (F_(2,25)_ = 0.095, η²p = 0.008, p = 0.909).

The graded score for free recall of words on Day 7 was insignificantly different among the CON (mean = 1.00), ExB4 (1.21) and ExAFTR (1.00) groups (F_(2,26)_ = 0.336, η²p = 0.025, p = 0.718). The ExB4 group was slightly better (d = 0.30) compared to CON. Across all three groups (n = 29), the grade scores were lower on Day 7 (1.1 ± 0.6) compared to Day 1 (1.4 ± 0.5). The Day 7 scores were insignificantly different across groups when using Day 1 scores as a covariate (F_(2,25)_ = 0.661, η²p = 0.050, p = 0.525).

The ANOVA for the total number of comprehensible sentences written on Day 7 was insignificantly different among the groups (F_(2,26)_ = 1.076, η²p = 0.076, p = 0.356). The ExAFTR wrote moderately fewer sentences compared to the CON (1.3 versus 2.1; d = −0.71). There was no difference between the CON and the ExB4 (2.1 vs.1.9 sentences) group (t_(15)_ = 1.391, p = 0.185). Across all three groups (n = 29), the number of comprehensible sentences was lower on Day 7 (1.8 ± 1.1) compared to Day 1 (2.6 ± 0.9). ANCOVA revealed that Day 7 scores were insignificantly different across groups when using Day 1 scores as a covariate (F_(2,25)_ = 1.058, η²p = 0.078, p = 0.362).

Recognition memory

Table 7 provides a summary of the long-term recognition memory results. Across all three groups (n = 29), recognition memory performance was better on Day 1 compared to Day 7 for hits (90.8 ± 14.9% vs 88.6 ± 14.6% accuracy). The percentage of words accurately recognized (hits) on Day 7 was insignificantly different among the CON (86.0%), ExB4 (95.7%) and ExAFTR (86.7%) groups (F_(2,26)_ = 1.108, η²p = 0.079, p = 0.345). The ExB4 group had moderately better recognition (d = 0.76) compared to CON. ANCOVA revealed that Day 7 scores were insignificantly different across groups when using Day 1 scores as a covariate (F_(2,25)_ = 0.775, η²p = 0.058, p = 0.471).

To facilitate comparisons, Table 9 provides a single source summary of Cohen’s d effect size differences between the exercise groups and CON for all the free-recall and recognition memory outcomes.

**Table 9:** Summary of Cohen’s d mean effect size differences between the exercise and control conditions for the free-recall and recognition memory outcomes.

	ExB4 vs. CON ES 95% CI	ExAFTR vs. CON ES 95% CI
Day 1		
**Free-recall**		
Discernable words	Favors exercise, d = 0.44; 95% CI = −.53, 1.42	Favors control, d = −0.27; 95% CI = −1.11, 0.57
Accurate words	Favors exercise, d = 0.28; 95% CI = −0.69, 1.25	Trivial difference
Grade score	Trivial difference	Trivial difference
Sentences	Trivial difference	Favors control, d = −0.99; 95% CI = −1.87, −0.09
**Recognition**	Favors exercise, d = 0.51; 95% CI = −0.47, 1.49	Favors control, d = −0.51; 95% CI = −1.36, 0.35
		
Day 7		
**Free-recall**		
Discernable words	Favors exercise, d = 0.25 95% CI = −0.72, 1.21	Trivial difference
Accurate words	Favors exercise, d = 0.21;-95% CI = 0.76, 1.18	Trivial difference
Grade score	Favors exercise, d = 0.30; 95% CI = −0.70, 1.27	Trivial difference
Sentences	Trivial difference	Favors control, d = −0.71; 95% CI = −1.70, 0.29
**Recognition**	Favors exercise, d = 0.76; 95% CI = −0.24, 1.76	Trivial difference

## 4. Discussion

### 4.1. Primary finding

Based on effect size comparisons to a non-exercise control, the primary finding of this investigation is that in a sample of older adolescents with ADHD – brief, high intensity cycling exercise prior to an initial Portuguese lesson was associated with a pattern of numerically higher free-recall and recognition memory for Portuguese words. These differences were not statistically significant and require confirmation in larger studies assessing causal mechanisms. The findings are consistent with high intensity exercise prior to the Portuguese lesson altering relevant brain neurochemicals, such as neurotransmitters (e.g., norepinephrine) and neurotrophic factors (e.g., brain-derived neurotropic factor), hypothesized to improve memory performance in part by enhancing attentional control processes and strengthening hippocampal encoding [31]. These findings also are inconsistent with prior experiments with children and young adolescents with ADHD reporting trivial effects of exercise on memory [9–11]. Possible explanations for the different study results include the absence of a no-exercise control group in one prior study [11], different cognitive task types (spatial and picture sequence memory versus vocabulary used here) [9,10], differences in age (8–14 vs. 18–21 years here), differences in exercise type, intensity and location (exergaming at home vs. laboratory cycling used here) [9] and differences in exposure to the language stimuli (<6 seconds compared to 29 total minutes used here).

When exercise was performed after the Portuguese lesson, twice as many trivial differences on the free-recall and recognition tests were found compared to exercise before the lesson. Also, no positive effects emerged while some negative effects did, with the largest being for sentence production. The results for sentence production were based on a post-hoc t-test following an insignificant ANOVA. This observation identified a potentially meaningful effect in this underpowered exploratory study. Consequently, the result should be viewed as a potential signal that merits consideration in future research rather than as conclusive evidence. Sentence production is the most complex task asked of the participants and it is known to involve several levels of cognitive processing and there is a lack of agreement about how it occurs with multiple models competing for primacy [32]. The present findings imply that exercise performed after a 2LL lesson in young adolescents with ADHD either disrupted these incompletely understood processes and/or, alternatively, reduced motivation to complete this more complex task [33]. Also, recognition memory on Day 1 was approximately one-half of a standard deviation when exercise was performed after the language lesson. This observation should be interpreted cautiously as it was not statistically significant and it ran counter to our hypothesis that, compared to a resting control condition, recognition memory performance would be best in the “Exercise After” condition. Our hypothesis was based on studies of neurotypical children, adolescents and adults which may have accounted for the different outcome given the ADHD sample tested here [34].

### 4.2. Manipulation check

While the exercise stimuli used here may have been inadequate to induce larger effects, (for example, more sprints might have larger effects), there is strong evidence that the exercise protocol used was effective. Participants exhibited typical perceptual and physiological responses to the seated resting control and the brief all-out cycling conditions. In short, these data confirm that the exercise manipulation generated clear differences between the CON and exercise groups. On average, the ExB4 group reported a greater perceptual effort (mean = 15.9, closest verbal anchor “hard”) compared to the ExAFTR group (mean = 13.5, closest verbal anchor “somewhat hard”). It is possible that this perceptual difference, likely based in part on differences in the degree to which the central nervous system was activated [35,36], contributed to variation in the Portuguese language learning results – even in the absence of significant group differences in heart rate during exercise.

### 4.3. State anxiety

In contrast to many prior laboratory investigations reporting state anxiety reductions after either quiet seated rest [37] or exercise [38], state anxiety did not change significantly in response to either the CON or the exercise conditions. Most of the prior research on this topic has involved continuous, longer duration (≥ 20 minutes) and low-to-moderate intensity exercise that did not involve sustained cognitive effort between the pre- and post-test state anxiety assessments. Also, state anxiety levels during the high intensity cycle sprints, while not measured here, plausibly increased and thereby contributed to a failure to detect an anxiety reduction – especially given that there was only one assessment timed immediately after the final sprint [39].

### 4.4. Limitations

The present investigation had several limitations. We cannot rule out that the findings were influenced by small sample bias and low statistical power was a limitation based on the sample sizes per group. A post-hoc statistical power analysis using G*power revealed that the present sample size of 29 was enough to detect only very large effect sizes (ANOVA f ≥ 0.61). Accordingly, the modest effect size differences found here were not statistically significant based on the conventional probability level of P < 0.05. Given this, the paper has emphasized the magnitude of the effects observed over statistical significance. Given the novelty of the research, the effect sizes generated warrant attention for evaluating current research on exercise timing and in planning future studies. Indeed, these observations need to be independently replicated, preferably in diverse and larger samples permitting stronger traditional tests of the hypotheses; until then the findings should be interpreted cautiously because the results may be imprecise

The recognition memory test results may have been limited by ceiling effects. About 50% of the total participants achieved the maximum score for recognition memory on both Day 1 and Day 7. Had more difficult words to remember been used in the Portuguese lesson or had more lure words been used in the recognition tests, it is possible that different results would have emerged in part because ceiling effects were minimized.

It was a potential limitation that some unmeasured variables may have accounted for variation in the primary outcome measures. Individuals with ADHD have motivational deficits, for example, that may have varied across the groups [34]. This variable was unmeasured. Nevertheless, our use of random assignment theoretically minimized this potential confound. It is possible that participants thought that exercise before a Portuguese lesson would improve their memory and that similar expectations were not harbored by those who completed exercise after the lesson. These types of expectations were not assessed. At least one prior investigation focused on the potential role of expectations in cognitive changes in response to acute exercise concluded that expectation-driven placebo effects are unlikely to account for cognitive response differences between exercise and control groups [40]. Other limitations include that neither 2LL performance or cognitive function were assessed comprehensively and it is plausible that larger or smaller exercise effects could have emerged had different methods been used. For example, the high overall performance in recognition memory (potential ceiling effect) in the present experiment could have masked larger exercise-induced differences in memory that could have otherwise emerged with a more challenging task. More generally, in the future our knowledge in this area will benefit from incorporating learning assessment batteries that include both multiple aspects of second language acquisition (e.g., reading, listening, speaking and writing comprehension) and larger samples (to avoid well known limitations associated with sub-optimal statistical power).

## 5. Conclusion

The findings from this exploratory study suggest that in a sample of older adolescents with ADHD: (i) brief, intense cycling exercise prior to an initial Portuguese lesson is associated with a pattern suggesting higher free-recall and recognition memory for Portuguese words while (ii) similar exercise performed after the language lesson is associated with a pattern suggesting worse memory performance on sentence production, a more complex cognitive task.
